# Body Mass Index and Employment-Based Health Insurance

**DOI:** 10.1186/1472-6963-8-101

**Published:** 2008-05-09

**Authors:** Ronald L Fong, Peter Franks

**Affiliations:** 1 Department of Family & Community Medicine, University of California, Davis, Sacramento, CA 95817, USA

## Abstract

**Background:**

Obese workers incur greater health care costs than normal weight workers. Possibly viewed by employers as an increased financial risk, they may be at a disadvantage in procuring employment that provides health insurance. This study aims to evaluate the association between body mass index [BMI, weight in kilograms divided by the square of height in meters] of employees and their likelihood of holding jobs that include employment-based health insurance [EBHI].

**Methods:**

We used the 2004 Household Components of the nationally representative Medical Expenditure Panel Survey. We utilized logistic regression models with provision of EBHI as the dependent variable in this descriptive analysis. The key independent variable was BMI, with adjustments for the domains of demographics, social-economic status, workplace/job characteristics, and health behavior/status. BMI was classified as normal weight (18.5–24.9), overweight (25.0–29.9), or obese (≥ 30.0). There were 11,833 eligible respondents in the analysis.

**Results:**

Among employed adults, obese workers [adjusted probability (AP) = 0.62, (0.60, 0.65)] (*P *= 0.005) were more likely to be employed in jobs with EBHI than their normal weight counterparts [AP = 0.57, (0.55, 0.60)]. Overweight workers were also more likely to hold jobs with EBHI than normal weight workers, but the difference did not reach statistical significance [AP = 0.61 (0.58, 0.63)] (*P *= 0.052). There were no interaction effects between BMI and gender or age.

**Conclusion:**

In this nationally representative sample, we detected an association between workers' increasing BMI and their likelihood of being employed in positions that include EBHI. These findings suggest that obese workers are more likely to have EBHI than other workers.

## Background

Employee health benefits are among the highest employer expenses. In the past two decades, employment-based health insurance (EBHI) premiums have increased nearly 10% per year [1]. Employee absenteeism due to illness further escalates employer expenditures; the annual cost of health-related lost production time approached $226 billion in 2002 [1]. Consequently, employers have tabbed employee health as a cost containment priority [2-5].

The prevalence of overweight adults has markedly increased in the last two decades. In the National Health and Nutrition Examination Surveys [NHANES] of 1976–80, the prevalence was 32.3%. The prevalence rose to 32.7% in NHANES III [1988–94] and to 34.1% during the survey years of 1999–2002 [6]. The United States workforce experienced similar increases in overweight individuals during the same period. The overweight group currently constitutes the largest percentage of employees [7].

The association between employee weight distributions with health outcomes may draw employer attention. Compared with normal weight workers, overweight and obese ones compile greater rates of: absenteeism [8-12], occupational injuries [13], short-term disability [14], and self-reported unhealthy physical and mental days [15,16]. These patterns contribute to disproportionately higher shares of the total medical claims cost and greater health care utilization by overweight and obese workers [17-20]. Increasing body mass index (BMI) [weight in kilograms/square of height in meters] predicts higher mortality in insured populations [21].

Employee and employer preferences interact to drive EBHI into a key position regarding hiring practices. Workers rate health insurance as the most desired benefit [22,23]. Worker turnover increases costs while decreasing productivity; employers include health insurance in their compensation to attract and to retain highly productive workforces [22,24,25]. For employers, health insurance functions to maintain, without necessarily improving, employee health status [26].

Based on prior studies documenting the financial and health risk of overweight and obese workers, we hypothesized that these workers are less likely than normal weight workers to obtain employment that includes health insurance. Such a relationship would be of concern because EBHI is the main source of health insurance for persons under 65 years of age [22,27-29]. Reduced coverage of workers via their employers increases the uninsured pool and, subsequently, increases the burden of public funding for their health needs [30]. These implications become further magnified as reports have projected continuing increases in BMI among workers [7]. Given these dynamics, we wanted to evaluate the degree and direction of the association of employees' BMI and their likelihood of obtaining EBHI.

## Methods

We utilized data from the 2004 Household Components (HC) of the Medical Expenditure Panel Survey (MEPS). The HC's survey frame is derived from the National Health Interview Survey (NHIS). NHIS is representative of the non-institutionalized civilian United States population and systematically over samples Hispanics and African Americans. The HC collects data including demographics, health conditions/status, health care utilization, health insurance coverage, income, employment status, workplace/job variables (number of employees at work site, blue/white collar occupation, union status, and hours worked per week). The HC employs an overlapping panel design, consisting of six interview rounds over a two-and-a-half year period, and a self-administered questionnaire (SAQ). Further details about the survey are available on the MEPS website [31]. The HC survey provides weights to determine population estimates of characteristics of survey respondents.

**Figure 1:**
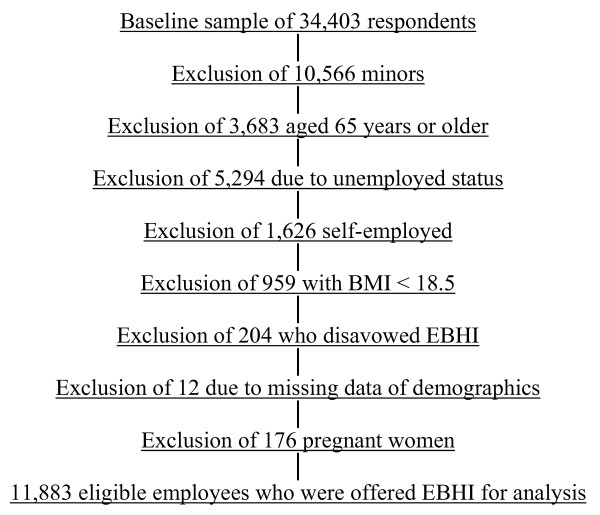
Selection criteria for inclusion in full model analysis.

The key dependent variable was holding a job that included EBHI, defined by a "yes" response to the query of "offered health insurance by current main job." The key independent variable was BMI, categorized by the National Institutes of Health classifications [32]: normal weight (18.5 – < 25), overweight (25 – < 30), and obese (≥ 30). We excluded individuals who were: underweight (BMI < 18.5) due to their increased risks for malignancies and eating disorders, pregnant, ≥ 65 years of age due to their eligibility for Medicare, or self-employed. Figure 1 details the inclusion criteria for analysis.

### Covariates

We categorized family income based on the following percentages of the Federal Poverty Level: low (≤ 200%), medium (≥ 200% and < 400%), and high (≥ 400%). Blue/white collar status was defined by the United States Census Occupational Codes.

### Analyses

We utilized STATA software, version 8.2 (StataCorp, College Station, Texas), for statistical analyses. Our analysis adjusted for the complex sampling strategy of MEPS to yield appropriate standard errors and nationally representative parameter estimates. We designated holding a job that included EBHI as the dependent variable in the logistic regression analyses. The key independent variable was BMI with adjustments for: demographics (age, race/ethnicity, sex, marital status, region of residency), whether a spouse held a job that included EBHI, the number of dependents in the household, excluding spouses, socio-economic status (SES) (income, years of completed education), workplace/job variables (full/part-time, union status, number of employees at work site, and blue/white collar occupation), and smoking status. We also analyzed the association of BMI with the likelihood of individuals enrolling in their EBHI plans, including all the covariates outlined above. To facilitate more meaningful interpretation of the logistic regression analyses, we reported parameter coefficients as adjusted probabilities, rather than odds ratios [33].

We evaluated the interaction effects between BMI and the salient independent variables of age and gender to explore possible modifications of the association between BMI and the likelihood of being offered EBHI. These analyses were based on the following considerations: health care expenditures associated with obesity are progressively higher with advancing age and among whites [34]; increasing age in overweight and obese individuals correlates with lower SES [35]; and overweight/obese women but not men tend to have lower SES and employment opportunities than their normal weight counterparts [36]. We also analyzed the data stratified by gender and observed similar results for both sexes. Our analysis did not detect any significant interactions between BMI and age or gender with respect to likelihood of being offered EBHI.

Thus, we present the results of combined female and male analysis.

## Results

Significant demographic differences existed among the three weight groups: overweight and obese workers tended to be older, married or have been married, and have greater Hispanic and African American representation. There were no differences among the groups with respect to the likelihood of having a spouse who held a job that included EBHI. Obese employees tended to be the least educated (Table 1). All three groups had slightly more than 20% smokers.

**Table 1:** Baseline characteristics of the study population

**BMI range, in kg/m^2^**	**18.5–24.9** **(n = 4,243)**	**25.0–29.9** **(n = 4,278)**	**≥ 30.0** **(n = 3,362)**
**Mean age, years (SE)**	37 (0.2)	41 (0.2)	42 (0.2)
**Gender**			
female (n = 5,743)	60 (1.0)	40 (0.7)	51 (0.9)
male (n = 6,140)	40 (1.0)	60 (0.7)	49 (0.9)
**Race/ethnicity, % (SE)**			
white	69 (1.1)	67 (1.1)	64 (1.3)
Hispanic	11 (0.7)	15 (0.8)	14 (0.9)
African American	10 (0.7)	12 (0.7)	18 (1.0)
other	10 (0.7)	6 (0.5)	5 (0.5)
**Marital status, % (SE)**			
never	35 (1.0)	25 (0.8)	24 (1.0)
married	52 (1.1)	59 (1.0)	59 (1.1)
widowed/divorced	13 (0.7)	15(0.7)	17 (0.9)
**Spouse offered EBHI, % (SE)**			
yes	26 (0.9)	28 (1.0)	27 (1.0)
no	74 (0.9)	72 (1.0)	73 (1.0)
**Mean number of dependents, (SE)**	1.30 (0.03)	1.37 (0.03)	1.37 (0.03)
**Family income, % (SE)**			
low	19 (0.8)	19 (0.8)	22 (0.9)
medium	33 (1.1)	32 (1.0)	39 (1.1)
high	48 (1.2)	49 (1.2)	39 (1.3)
**Completed years of education, % (SE)**			
<12	13 (0.6)	14 (0.7)	15 (0.7)
= 12	28 (1.0)	31 (1.0)	36 (1.1)
>12	59 (1.2)	55 (1.2)	49 (1.2)
**Metropolitan area residency, % (SE)**			
yes	85 (1.3)	84 (1.3)	83 (1.3)
no	15 (1.3)	16 (1.3)	17 (1.3)
**Region of residency, % (SE)**			
northeast	20 (1.0)	19 (1.0)	16 (1.1)
midwest	24 (1.2)	23 (1.3)	23 (1.3)
south	33 (1.6)	36 (1.4)	41 (1.5)
west	23 (1.4)	22 (1.2)	20 (1.2)

Notes: Percentages and means are based on weighted and unadjusted data. *P *values for overall group comparison based on χ^2 ^tests. All *P *values <0.001, except age between overweight and obese (<0.05). Differences in number of dependents between overweight and obese were non-significant. Differences among groups based on metropolitan area residency and whether spouse held a job with EBHI were non-significant. Column totals may not add up to 100 due to rounding.

Employee characteristics associated with an increased likelihood of holding a job with EBHI included: being obese, age > 40 years, higher SES, having been married at any time, and having a spouse who did not have access to EBHI. Among ethnic groups, Hispanics were the least likely to be employed in jobs with EHBI. Employees in jobs that included EBHI were more likely to be employed full-time, be a union member, and work at a location with more than 25 employees (Table 2).

**Table 2:** Relationship between whether employees held jobs with employment-based health insurance (EHBI) with other characteristics

**Held jobs with EBHI**	**Yes % (SE)**	**No % (SE)**
**BMI group**		
normal	34 (0.8)	42 (1.1)
overweight	38 (0.8)	35 (0.9)
obese	28 (0.7)	24 (0.8)
**Age, years**		
< 40	46 (0.9)	58 (0.9)
≥ 40	54 (0.9)	42 (0.9)
**Race/ethnicity**		
white	71 (0.9)	65 (1.3)
Hispanic	10 (0.6)	17 (1.0)
African American	12 (0.7)	10 (0.7)
other	7 (0.5)	7 (0.5)
**Marital status**		
never	26 (0.8)	33 (0.9)
married	58 (0.9)	54 (0.9)
widowed/divorced	16 (0.6)	13 (0.6)
**Spouse offered EBHI**		
yes	24 (0.8)	31 (0.9)
no	76 (0.8)	69 (0.9)
**Number of dependents**		
0	39 (0.8)	29 (0.8)
1	23 (0.7)	21 (0.8)
2	21 (0.7)	24 (0.8)
3	11 (0.5)	14 (0.6)
4 or more	6 (0.4)	11 (0.7)
**Family income**		
low	13 (0.5)	32 (1.0)
medium	35 (0.9)	32 (1.0)
high	52 (1.0)	36 (0.7)
**Completed years of education**		
<12	9 (0.4)	22 (0.9)
= 12	30 (0.8)	34 (1.0)
>12	62 (1.0)	43 (1.2)
**Metropolitan area residency**		
No	15 (1.1)	18 (1.5)
Yes	85 (1.1)	82 (1.5)
**Region of residency**		
northeast	19 (0.9)	18 (1.0)
midwest	23 (1.0)	24 (1.3)
south	35 (1.4)	38 (1.5)
west	22 (1.0)	21 (1.2)
**Smoking status**		
yes	20 (0.6)	25 (0.8)
no	80 (0.6)	75 (0.8)
**Full-time employment**		
yes	93 (0.4)	55 (1.0)
no	7 (0.4)	44 (1.0)
**Number of employees at workplace**		
Not ascertained/did not know	5 (0.4)	15 (0.8)
1–10	12 (0.5)	29 (0.9)
11–25	12 (0.5)	17 (0.8)
26–100	26 (0.7)	21 (0.8)
> 100	45 (0.9)	18 (0.8)
**Union member**		
yes	18 (0.8)	4 (0.4)
no	81 (0.7)	96 (0.5)
**Blue collar**		
yes	25 (0.8)	24 (0.9)
no	75 (0.8)	76 (0.9)

Notes: Percentages are based on weighted and unadjusted data. *P *values for overall group comparison based on χ^2 ^tests. All *P *values <0.001, except region of residency and blue collar status, which were not significant. Column totals may not add up to 100 due to rounding.

The results of the adjusted analysis examining holding jobs with EBHI and subsequent plan enrollment among workers are shown in Table 3. Obese workers were more likely to hold jobs with EBHI than normal weight workers. Overweight workers tended to be more likely to hold jobs with EBHI than normal weight workers, but the difference did not reach statistical significance (*P *= 0.052). When we classified BMI as a continuous variable in the regression analysis, the β coefficient was 0.015 (95% confidence intervals 0.006, 0.024, *P *= 0.002). Of those who enrolled in EBHI plans, there were no differences among the three groups. The adjusted odds ratios for the covariates in the regression model analyzing holding jobs with EBHI are listed in Table 4.

**Table 3:** Adjusted probability (95% CI) of workers being offered and holding employment-based health insurance (EBHI) if offered, by BMI

**BMI range, in kg/m^2^**	**18.5–24.9**	**25.0–29.9**	**>30.0**
Offered EBHI (n = 11,883)	0.57 (0.55, 0.60)	0.61 (0.58, 0.63)	0.62* (0.60, 0.65)
Held EBHI (n = 6,792)	0.91 (0.92, 0.95)	0.94 (0.93, 0.95)	0.92 (0.90, 0.94)

*P = 0.005 when compared to normal weight group.

**Table 4:** Adjusted Odds ratios [OR] for covariates listed in regression model with confidence intervals [CI]

**Covariate**	**OR**	**95% CI**	***P***
**BMI**			
normal*	1.00		
overweight	1.16	1.00, 1.34	0.052
obese	1.23	1.07, 1.43	0.004
**Age, per year**	1.00	1.00, 1.01	0.166
**Race**			
white*	1.00		
Hispanic	0.69	0.57, 0.85	<0.001
African American	1.20	1.00, 1.45	0.053
other	0.94	0.74, 1.21	0.647
**Gender**			
female*	1.00		
male	1.13	0.99, 1.28	0.067
**Family income**			
low*	1.00		
medium	2.11	1.83, 2.42	<0.001
high	2.08	1.78, 2.43	<0.001
**Years education**			
<12*	1.00		
= 12	1.74	1.46, 2.07	<0.001
>12	2.47	2.01, 3.04	<0.001
**Marital status**			
never married*	1.00		
widowed/divorced/separated	1.15	0.94, 1.41	0.167
married	1.26	1.05, 1.51	0.014
**Number of dependents, excluding spouse**			
none*	1.00		
one	0.87	0.75, 1.01	0.070
two	0.70	0.60, 0.82	<0.001
three	0.83	0.69, 0.99	0.035
four or more	0.63	0.50, 0.81	<0.001
**Smoker**			
no*	1.00		
yes	0.88	0.77, 1.00	0.043
**Metropolitan area**			
no*	1.00		
yes	1.01	0.89, 1.16	0.833
**Region of residency**			
northeast*	1.00		
midwest	0.91	0.75, 1.10	0.314
south	1.02	0.84, 1.25	0.818
west	1.23	1.01, 1.50	0.040
**Number of prescription medications**			
none*	1.00		
1 to 2	1.14	0.97, 1.35	0.112
3 to 5	1.37	1.13, 1.66	0.001
4 to 14	1.37	1.17, 1.61	<0.001
15 or more	1.38	1.15, 1.65	<0.001
**Blue collar employment classification**			
no*	1.00		
yes	1.02	0.89, 1.17	0.814
**Union membership**			
no*	1.00		
yes	3.55	2.72, 4.63	<0.001
**Employment status**			
part-time*	1.00		
full-time	7.19	6.20, 8.33	<0.001
**Number of employees at workplace**			
unknown*	1.00		
1 to 10	0.77	0.61, 0.97	0.029
11 to 25	1.29	1.01, 1.65	0.045
26 to 100	1.91	1.49, 2.44	<0.001
101 or more	3.30	2.60, 4.18	<0.001
**Spouse offered EBHI**			
no*	1.00		
yes	0.34	0.28, 0.41	<0.001

*Reference category.

## Discussion

No prior studies have examined the relationship between employees' BMI and their likelihood of holding jobs that included EBHI. Our analysis of a nationally representative health survey demonstrated significant differences between obese and normal weight workers in their likelihood of having EBHI. These differences between overweight workers and normal weight workers approached, but did not obtain, statistical significance. When we evaluated BMI as a continuous variable, our results showed a direct association between increasing BMI and the increased likelihood of holding a job with EBHI; heavier workers were more likely to have jobs that included EBHI. This finding may alter the way employers utilize health insurance as a competitive benefit in the market for desired workers.

We predicted that the greater financial risk to employers posed by obese workers would result in those workers having reduced access to jobs with EBHI. Our findings are counter to those predicted. There may be several explanations for our findings. First, the results may reflect the effects of the differences in health status between normal weight workers and their obese counterparts. Previous studies have documented a linear relationship between increasing BMI and increasing health care expenditures [5,37,38]. Obesity is associated with a negative impact on self-rated health among adults, in the absence of and adjusting for chronic disease states [39]. Obese workers may have an increased concern and awareness of their health status and actively seek means to address those concerns.

Our previous work demonstrated that obese patients were more likely to be satisfied with their patient care experience than normal weight patients [40]. The variables associated with the health status domain accounted for the largest modification in the BMI-patient satisfaction relationship. This increased satisfaction would be consistent with an enhanced value placed on medical care by obese individuals. Berger, et al. [26] have proposed a model that has health status as the axis for the interface between an employee's well-being and an employer's demand for maximum workplace productivity. Employers' primary concerns about the employee are limited to the workplace. However, employees' investments into their jobs are balanced by the values placed on employment and non-work activities. Overweight and obese workers' emphasis on health may shift the investment of their efforts to seeking employment that offers health insurance.

Employers should be cognizant of the increasing BMI of their workforce and the value of providing health insurance to their employees. This becomes a shared venture since employees are also contributing to the cost of health insurance. Individuals with health insurance are more likely to utilize more health services [41] and receive more preventative care [42]. Overweight and obese patients with EBHI pose a substantial financial investment to the employers. Perhaps, employers can place a greater emphasis on obesity prevention through their health plans in an effort to increase the value of their health insurance expenditure. This may be a point of further collaboration between employer and employee to decrease costs and improve health outcomes while making accessibility to health insurance a priority.

Limitations of this study include its cross-sectional nature, the use of self-reported height and weight, and the lack of employment duration. The sequence of events is difficult to delineate in a cross-sectional analysis. With the passage of time, employees gain seniority and opportunities for pay increase and benefit eligibility. An employee may have obtained EBHI and subsequently gained weight with age. Our lower range for the normal BMI category of 18.5 may not have been sensitive enough to capture individuals with illness that would limit, but not eliminate, opportunities for employment and EBHI. In analyses not presented, we adjusted our normal BMI category to 20–24.9 to account for this potential bias and found no significant difference from the original analysis. MEPS collects data on an individual's yearly total medical expenses, but does not separate co-payment and deductible amounts. Accordingly, we are unable to determine if any association exists between these variables and the actions of employees in pursuing EBHI.

## Conclusion

With a significant percentage of their insured workforce overweight or obese, coupled with rising health care costs, employers may engage employees more directly on health issues. Currently, most employers view obesity as a matter of individual accountability [43]. Should employers shift their classification of obesity as a chronic disease as opposed to a lifestyle behavior, they may become more involved with their employees' health and coverage for chronic conditions in general. These interactions may include implementing preventive health services through on-site programs to reduce morbidity and costs [44], or initiating discussions with employees centering on health plan options [29]. Employees with EBHI are less likely to miss work and have fewer missed work days [45]. Employees who self-report poor health are more interested in having access and guidance from health care consultants and are more willing to follow through with action plans than those who self-report good health [46]. Thus, the targeted group for these employer innovations may be the most motivated and responsive.

## Competing interests

The authors declare that they have no competing interests.

## Authors' contributions

RLF conceived of the study, participated in the design of the study, performed the statistical analysis, and prepared the manuscript. PF participated in the design of the study, analyzed and interpreted the data, and provided editorial revisions of the manuscript.

## Pre-publication history

The pre-publication history for this paper can be accessed here:


